# Acute effects of caffeine and paracetamol on velocity and power in resistance exercise

**DOI:** 10.3389/fphys.2025.1536591

**Published:** 2025-02-24

**Authors:** Bela Scapec, Jozo Grgic, Dorian Varovic, Pavle Mikulic

**Affiliations:** ^1^ Faculty of Kinesiology, University of Zagreb, Zagreb, Croatia; ^2^ NUS Academy for Healthy Longevity, Yong Loo Lin School of Medicine, National University of Singapore, Singapore, Singapore

**Keywords:** ergogenic aids, medication, pain relief, interactions, exercise

## Abstract

This study aimed to explore the isolated and combined effects of caffeine and paracetamol (acetaminophen) on velocity and power in resistance exercise. Twenty-eight resistance-trained men and women participated in this randomized, double-blind, placebo-controlled, crossover study. The participants performed three sets of the bench press with 75% of one-repetition maximum to momentary muscular failure after ingesting a placebo, caffeine (3 mg/kg), paracetamol (1,500 mg), or caffeine + paracetamol 45 min before exercise. Velocity and power of the repetitions in each set were analysed. Compared to placebo, only isolated caffeine ingestion increased mean velocity (*d* = 0.34), and mean power (*d* = 0.17) in the first set. No significant differences between the conditions were observed for any of the analysed outcomes in the second and third sets. Our results indicate that only isolated caffeine ingestion improves velocity and power in resistance exercise, even though these effects are not consistent across all sets. Paracetamol ingestion was not ergogenic, even when combined with caffeine. These results highlight that analgesics may be ineffective in improving resistance exercise performance.

## Introduction

Caffeine is a well-established ergogenic aid, with research also exploring its effects on resistance exercise performance (Grgic, 2021). Evidence indicates that caffeine ingestion may enhance muscular strength, endurance, power, and velocity in resistance exercise (Grgic, 2021). While the exact mechanisms by which caffeine exerts its ergogenic effects are not yet fully clear, they are generally explained by caffeine’s ability to bind to adenosine receptors, which may reduce perceived exertion/pain and improve performance (McLellan et al., 2016; Guest et al., 2021). While the ergogenic effects of caffeine are well explored, other pharmacological agents such as analgesics, have been much less examined.

One of the most consumed analgesics is paracetamol (acetaminophen). There is a theoretical basis as to why analgesics such as paracetamol may be ergogenic for resistance exercise performance. Paracetamol consumption has been shown to attenuate the decline in electromyography occurring during repeated maximum isometric contractions, which might occur due to the paracetamol-induced increase in muscle activation (Morgan et al., 2018). Paracetamol is also effective in decreasing pain perception as it inhibits prostaglandin synthesis, which reduces the transduction of the sensory nerves and decreases nociceptive impulse transmission (Graham et al., 2013). These effects are important to consider for resistance exercise performance given that sets performed to muscular failure with 30% and 80% of one-repetition maximum (1RM) result in considerable increases in perceived exertion and pain levels (Lixandrão et al., 2019). Therefore, reducing resistance exercise-induced pain via the consumption of paracetamol may produce an ergogenic effect (Grgic, 2022).

Caffeine and paracetamol are commonly combined as they produce additive effects on pain relief (Derry et al., 2012). When consumed in isolation, caffeine and paracetamol are reported to enhance performance, but less is currently known about the effects that occur with their co-ingestion (Jessen et al., 2021; Tomazini et al., 2020). Exploring their co-ingestion is important as many athletes ingest more than one dietary supplement, and some use analgesics to improve their performance (Burke, 2017; Küster et al., 2013). Given that caffeine and paracetamol may be acting through similar mechanisms, it is plausible that their co-ingestion might result in additive effects on exercise performance. In this *Brief Research Report*, we explored the isolated and combined effects of caffeine and paracetamol on velocity and power in resistance exercise. We hypothesized that both caffeine and paracetamol would enhance performance.

## Methods

### Study design

In this randomized, double-blind, placebo-controlled, crossover design, the participants attended five sessions—one familiarization and four experimental sessions. All testing sessions were conducted in the morning hours (7–9 a.m.), and at the same time for each participant to avoid the effects of circadian variation. Each participant was instructed to follow their standard sleep routine before the testing sessions. The experimental trials took place four to 7 days apart. In the 24 h before every testing session, the participants were instructed to refrain from performing strenuous exercise. The first session included 1RM testing in the bench press and familiarization with the testing protocol. The remaining sessions were performed in a randomized and counterbalanced order in which participants ingested either caffeine, paracetamol, caffeine + paracetamol, or placebo. The participants consumed gelatine capsules that contained either caffeine (3 mg/kg), paracetamol (1,500 mg), placebo (1,000 mg maltodextrin), or caffeine + paracetamol in their respective doses. Previous studies reported that these doses are ergogenic and are associated with minimal side effects (McLellan et al., 2016; Grgic, 2022; Grgic and Mikulic, 2021). Substances for all conditions were provided in capsules, which were of identical appearance. In the experimental trials, the participants reported to the laboratory after an overnight fast and consumed one banana prior to ingesting the capsules, to reduce the likelihood of side effects associated with fasted paracetamol consumption (Whitcomb and Block, 1994). The participants were instructed to keep their nutritional habits the same throughout the study duration. The capsules were ingested 45 min before the start of the testing session. Experimental sessions consisted of performing three sets of the bench press exercise with 75% of 1RM to momentary muscular failure.

### Participants

This study included a sample of resistance-trained men and women as participants. To be eligible for the study, participants had to possess the ability to lift 100% and 60% of their body mass in the bench press for men and women, respectively (Santos et al., 2021). They were also required to have a minimum of 1 year of resistance training experience and be 18–45 years old. The exclusion criteria were: (a) the existence of any health limitations; (b) prior use of anabolic steroids; (c) contraindications pertaining to caffeine and/or paracetamol use. Initially, 35 prospective participants were recruited. Six did not complete the experimental trials (lost interest or personal reasons; sustained an injury outside the study), and one had data missing due to faulty equipment. Thus, a total of 28 participants completed all experimental sessions (Table 1). The Committee for Scientific Research and Ethics of the University of Zagreb Faculty of Kinesiology provided ethical approval for the study (approval number 28/2023; document dated 3 April 2023; Scapec et al., 2024). All participants were informed of the potential risks and benefits of the study and signed an informed consent form before enrolling in the study.

**TABLE 1:** Participants characteristics.

Variable	Whole sample (*n* = 28)	Females (*n* = 17)	Males (*n* = 11)
Age (years)	25 ± 4	25 ± 3	24 ± 5
Height (cm)	173 ± 9	167 ± 5	182 ± 2
Body mass (kg)	71 ± 11	64 ± 6	81 ± 10
1RM (kg)	70 ± 29	50 ± 9	100 ± 20

Data are presented as mean ± standard deviation; 1RM: one repetition maximum.

### 1RM testing

The first session included 1RM testing and familiarization with the exercise protocol. The protocol started with a self-selected warm-up lasting 10 min that consisted of various dynamic movements (e.g., arm swings, internal or external rotations of the shoulder joint, arm abduction or adduction). In addition, participants performed a specific warm-up routine, working up to their 1RM. The first warm-up set included 8–10 repetitions with a load set to 20 kg (empty Olympic barbell). The second, third, and fourth warm-up sets included 8–10, 3–6, and 1 repetition with 50, 75, and 95% of their estimated 1RM, respectively. Following the warm-up sets, the participants progressively increased the load (in consultation with the assessor) until they reached their true 1RM. Three-minute rest intervals were provided between each attempt. An unsuccessful 1RM attempt was deemed when a participant could not complete the concentric portion of the repetition. All 1RM values were determined within five attempts. Only in the familiarization session a 5-min rest interval was provided between obtaining the final 1RM value and a single set with 75% 1RM to momentary muscular failure in the bench press exercise.

### Bench press

After arriving at the laboratory, the participants performed a self-selected 10-min warm-up, which they were instructed to keep consistent throughout the study. The type of warm-up activity was monitored to ensure uniformity across sessions. An additional specific bench press warm-up was performed using an empty Olympic barbell (20 kg), followed by 50% of 1RM for 8–10 repetitions. The participants were instructed on the proper technique and were required to perform the lift while maintaining five points of contact with the bench. In addition, they were instructed to perform the concentric portion of the lift with maximum effort and velocity; the eccentric portion lasted 1–2 s with no pause at the bottom phase (i.e., during contact with the pectoralis muscle) (Varovic et al., 2023). The participants performed three sets of the bench press exercise with 75% of 1RM to momentary muscular failure, with a rest interval of 3 min between the sets. In addition to the number of repetitions, we also collected kinetic data. We attached a GymAware linear position transducer (GymAware Power Tool, Kinetic Performance Technologies, Canberra, Australia) to the barbell—a valid and reliable method of measuring kinetic parameters (Grgic et al., 2020a). This device measured the concentric mean velocity (m/s), mean power (W), peak velocity (m/s), and peak power (W) for each repetition. We evaluated the velocity and power of the performed repetitions by matching the number of repetitions between the four experimental conditions. As an example, if a participant performed 10 repetitions during the caffeine trial, and 9 repetitions during three other conditions (i.e., placebo, paracetamol, caffeine + paracetamol), we only evaluated velocity and power performed in the first 9 repetitions in all conditions. With such an approach, we objectively evaluated the “quality” of performed repetitions, even when their overall quantity was the same (Grgic et al., 2020b).

### Assessment of blinding

To assess the efficacy of the blinding in the experimental trials (before and after the testing sessions), we asked the participants to respond to the following question: “Please state which treatment you think you have received?” (Saunders et al., 2017). The participants were able to choose one of five answers: (a) “paracetamol”; (b) “caffeine”; (c) “paracetamol + caffeine”; (d) “placebo; and (e) “I do not know.” If the participants selected answers (a), (b), (c), or (d), they were also required to state the reason for choosing their response.

### Statistical analysis

To compare the effects between the conditions for the performance outcomes (mean velocity, mean power, peak velocity, and peak power), we analysed the data from each of the three sets using a repeated measures analysis of variance (ANOVA). Dunnett’s *post hoc* test was performed in the case of a significant main effect from the ANOVA. In this *post hoc* test, the control condition (placebo) was compared with the three experimental conditions (caffeine, paracetamol, and caffeine + paracetamol). The level of statistical significance was set at *p* < 0.05. Cohen’s *d* for repeated measures was calculated and presented with 95% confidence intervals (95% CI). The interpretation of effect sizes was based on the following thresholds: <0.20 (trivial), 0.20 to 0.49 (small), 0.50 to 0.79 (moderate), and ≥0.80 (large). All analyses were performed using the STATISTICA software (version 14.1.0.8; TIBCO Software Inc. Paolo Alto, CA, United States).

## Results

### Bench press

In the 1. set, there was a significant main effect of condition for mean velocity (*p* = 0.024; Table 2). The *post hoc* comparisons indicated that caffeine consumption increased mean velocity (*p* = 0.010; Cohen’s *d* = 0.34; 95% CI = 0.07, 0.62). There were no significant differences for paracetamol (*p* = 0.719; Cohen’s *d* = 0.02; 95% CI = −0.30, 0.33) and caffeine + paracetamol (*p* = 0.244; Cohen’s *d* = 0.16; 95% CI = −0.09, 0.43). There was a significant main effect of condition for mean power (*p* = 0.027). The *post hoc* comparisons indicated that caffeine consumption increased mean power (*p* = 0.004; Cohen’s *d* = 0.17; 95% CI = 0.04, 0.31). There were no significant differences for paracetamol (*p* = 0.337; Cohen’s *d* = 0.06; 95% CI = −0.04, 0.16) and caffeine + paracetamol (*p* = 0.149; Cohen’s *d* = 0.09; 95% CI = −0.003, 0.20). There was no significant main effect for peak velocity (*p* = 0.254) or peak power (*p* = 0.439) and no *post hoc* analysis was performed.

**TABLE 2:** Summary of the performance data.

Variable	Placebo	Caffeine	Paracetamol	Caffeine + paracetamol
*1. set*				
Mean velocity (m/s)	0.35 ± 0.06*	0.37 ± 0.07*	0.35 ± 0.05	0.36 ± 0.07
Mean power (W)	177 ± 77*	192 ± 94*	182 ± 88	185 ± 85
Peak velocity (m/s)	0.53 ± 0.10	0.55 ± 0.13	0.52 ± 0.10	0.54 ± 0.11
Peak power (W)	312 ± 174	325 ± 202	308 ± 187	318 ± 192
*2. set*				
Mean velocity (m/s)	0.31 ± 0.05	0.31 ± 0.06	0.31 ± 0.05	0.31 ± 0.05
Mean power (W)	158 ± 74	162 ± 81	160 ± 73	161 ± 76
Peak velocity (m/s)	0.48 ± 0.11	0.49 ± 0.11	0.49 ± 0.11	0.49 ± 0.12
Peak power (W)	293 ± 186	296 ± 191	290 ± 195	297 ± 191
*3. set*				
Mean velocity (m/s)	0.30 ± 0.06	0.30 ± 0.05	0.29 ± 0.05	0.29 ± 0.06
Mean power (W)	155 ± 72	154 ± 69	149 ± 69	151 ± 71
Peak velocity (m/s)	0.48 ± 0.11	0.48 ± 0.11	0.46 ± 0.10	0.48 ± 0.12
Peak power (W)	280 ± 176	290 ± 187	274 ± 179	289 ± 188

Data are reported as mean ± standard deviation; * = significant difference between the conditions.

In the 2. set, there was no significant main effect for mean velocity (*p* = 0.729), mean power (*p* = 0.825), peak velocity (*p* = 0.707), or peak power (*p* = 0.886), and no *post hoc* analysis was performed. In the 3. set, there was no significant main effect for mean velocity (*p* = 0.483), mean power (*p* = 0.606), peak velocity (*p* = 0.281), or peak power (*p* = 0.330), and no *post hoc* analysis was performed.

### Blinding

When evaluated pre-exercise, the conditions were correctly identified by 2 (caffeine) or 3 participants (placebo, paracetamol, caffeine + paracetamol). None of these participants correctly identified all four conditions.

When evaluated post-exercise, the conditions were correctly identified by 5 (placebo, caffeine + paracetamol), 7 (caffeine), and 8 (paracetamol) participants. None of these participants correctly identified all four conditions.

## Discussion

The main finding of this study is that only isolated caffeine ingestion was effective in enhancing mean velocity and mean power. However, these effects were present only in the first set and were not observed in the second and third sets. Isolated paracetamol ingestion or its combination with caffeine was not ergogenic for any of the analysed outcomes.

An ergogenic effect of caffeine was found for mean velocity and mean power, but only in the first set (*d* = 0.17–0.34). Grgic et al. (2020b) used a similar design where 3 mg/kg of caffeine was provided before completing a single set of bench press with 85% of 1RM. In accordance with our findings, Grgic et al. (2020b) observed an ergogenic effect on mean velocity and mean power (*d* = 0.57–0.85). Collectively, it seems that caffeine is an effective ergogenic aid in improving the “quality” of completed repetitions, which is an important finding when placed in the context of the data from studies using velocity-based training (Galiano et al., 2022). While an ergogenic effect was found in the first set, no significant difference between the conditions was found for the second and third sets. Giráldez-Costas et al. (2020) provided 3 mg/kg of caffeine before an exercise session involving four sets of eight repetitions in the bench press at 70% of 1RM. Data indicated that caffeine ingestion increased mean and peak velocity and power in all four sets. These divergent findings between the studies may be explained by the differences in our methodological approaches. Giráldez-Costas and colleagues (2020) used a protocol where the participants performed a fixed number of repetitions each set (i.e., 8 repetitions), as they did not perform the exercise to the point of muscular failure. In our study, the participants performed each set to muscular failure, and kinetic variables were evaluated by matching the number of completed repetitions between the conditions. It might be that caffeine’s effects on velocity and power are more consistent when the exercise bout does not include performing sets to muscular failure. The combined ingestion of caffeine and paracetamol was not ergogenic, possibly because paracetamol counteracted some of the effects of caffeine. Pre-clinical data indicate that paracetamol reduces motivation, which may reduce caffeine’s ergogenic potential (Chen et al., 2018). However, we did not explore these specific outcomes, which is something that future studies may consider.

Isolated ingestion of paracetamol was not ergogenic for any of the analysed variables. Due to the limited evidence base, comparison of our results with other studies using paracetamol is limited. Only two studies (Morgan et al., 2018; Morgan et al., 2019) explored the effects of paracetamol on resistance exercise-related variables. One study reported that paracetamol ingestion (1,000 mg) may increase torque in a protocol involving 60 × 3-s maximum isometric contraction (Morgan et al., 2018). Another study evaluated time-to-task failure using an isokinetic protocol and reported that performance did not differ between the paracetamol and placebo trials (Morgan et al., 2019). Again, the design of these studies differs from ours, as we used a more traditional resistance exercise protocol. While there are no studies using such a protocol while providing paracetamol, data are available for other agents used for pain relief, such as ibuprofen (Correa et al., 2013). For example, Correa et al. (2013) included 12 participants who ingested either 1,200 mg of ibuprofen or placebo, 1 h before performing six sets of bench press and squats to muscular failure. The number of performed repetitions declined with each set, but there was no significant difference between the placebo and ibuprofen conditions. Overall, it seems that analgesics such as paracetamol and ibuprofen are ineffective in improving resistance exercise performance, even though future studies on the topic are still needed.

While this study has several strengths, such as the use of a double-blind design and a comprehensive evaluation of resistance exercise performance, there are also several limitations that need to be considered. We included a relatively large sample size, especially considering that the majority of previous studies on paracetamol and exercise performance were conducted in samples with less than 20 participants (Grgic, 2022). Still, when we conducted a *post hoc* power analysis, it indicated that our achieved power varied substantially. For example, it was only 5% for mean velocity in the first set when comparing paracetamol vs. placebo, likely because Cohen’s *d* here was 0.02. The power was also up to 90% when comparing the mean velocity of the first set between caffeine and placebo. Therefore, while we did include a relatively large sample size, it is possible that the study was underpowered to detect very small (or trivial) effects. We used a dose and timing of consumption based on previous studies reporting an ergogenic effect of paracetamol (Maguer et al., 2010; Maguer et al., 2014). However, the absolute dose provided to all participants resulted in having different relative doses (i.e., per kg of body mass), which might have confounded the results. We provided paracetamol 45 min before exercise as paracetamol plasma half-life is 1.5–2.5 h (Forrest et al., 1982; Prescott, 1980). Thus, it is likely that the participants performed the exercise test at, or close to, peak paracetamol plasma levels. Indeed, there is evidence that paracetamol peak plasma levels occur around 30–45 min post ingestion, even though there is between-individual variation (Prescott, 1980). However, a limitation of our study is that we did not evaluate paracetamol plasma levels, which should be explored in future studies. Future studies may consider incorporating a baseline assessment to evaluate time-to-peak paracetamol plasma levels and use an individualized approach when prescribing the timing of consumption pre-exercise, as has been done for sodium bicarbonate (Oliveira et al., 2020). Finally, while we instructed the participants to follow their normal sleep patterns before the experimental sessions, we did not collect data sleep-related data (e.g., sleep duration). This is a consideration that needs to be mentioned as sleep may moderate caffeine’s effect (Cook et al., 2012; Romdhani et al., 2022).

## Conclusion

In summary, our results indicate that only isolated caffeine ingestion improves velocity power in resistance exercise, even though these effects are not consistent across all sets. Isolated paracetamol ingestion was not ergogenic, even when combined with caffeine. These results highlight that analgesics may be ineffective in improving resistance exercise performance.

## Data Availability

The raw data supporting the conclusions of this article will be made available by the authors, without undue reservation.

## References

[B1] Burke L. M. (2017). Practical issues in evidence-based use of performance supplements: supplement interactions, repeated use and individual responses. Sports. Med. 47, 79–100. 10.1007/s40279-017-0687-1 28332111 PMC5371635

[B2] Chen Z. Wei H. Pertovaara A. Wang J. Carlson S. (2018). Anxiety- and activity-related effects of paracetamol on healthy and neuropathic rats. Pharmacol. Res. Perspect. 6, e00367. 10.1002/prp2.367 29417759 PMC5817821

[B3] Cook C. Beaven C. M. Kilduff L. P. Drawer S. (2012). Acute caffeine ingestion's increase of voluntarily chosen resistance-training load after limited sleep. Int. J. Sport. Nutr. Exerc. Metab. 22, 157–164. 10.1123/ijsnem.22.3.157 22349085

[B4] Correa C. S. Cadore E. L. Baroni B. M. Silva E. R. Bijoldo J. M. Pinto R. S. (2013). Effects of prophylactic anti-inflammatory non-steroidal ibuprofen on performance in a session of strength training. Rev. Bras. Med. Esporte. 19, 4.

[B5] Derry C. J. Derry S. Moore R. A. (2012). Caffeine as an analgesic adjuvant for acute pain in adults. Cochrane. Database. Syst. Rev. cd009281., CD009281. 10.1002/14651858.CD009281.pub2 22419343

[B6] Forrest J. A. Clements J. A. Prescott L. F. (1982). Clinical pharmacokinetics of paracetamol. Clin. Pharmacokinet. 7, 93–107. 10.2165/00003088-198207020-00001 7039926

[B7] Galiano C. Pareja-Blanco F. Hidalgo de Mora J. Sáez de Villarreal E. (2022). Low-velocity loss induces similar strength gains to moderate-velocity loss during resistance training. J. Strength. Cond. Res. 36, 340–345. 10.1519/JSC.0000000000003487 31904715

[B8] Giráldez-Costas V. González-García J. Lara B. Coso J. D. Wilk M. Salinero J. J. (2020). Caffeine increases muscle performance during a bench press training session. J. Hum. Kinet. 74, 185–193. 10.2478/hukin-2020-0024 33312286 PMC7706635

[B9] Graham G. G. Davies M. J. Day R. O. Mohamudally A. Scott K. F. (2013). The modern pharmacology of paracetamol: therapeutic actions, mechanism of action, metabolism, toxicity and recent pharmacological findings. Inflammopharmacology 21, 201–232. 10.1007/s10787-013-0172-x 23719833

[B10] Grgic J. (2021). Effects of caffeine on resistance exercise: a review of recent research. Sports. Med. 51, 2281–2298. 10.1007/s40279-021-01521-x 34291426

[B11] Grgic J. (2022). What is the effect of paracetamol (acetaminophen) ingestion on exercise performance? Current findings and future research directions. Sports. Med. 52, 431–439. 10.1007/s40279-021-01633-4 35038139

[B12] Grgic J. Mikulic P. (2021). Effects of paracetamol (acetaminophen) ingestion on endurance performance: a systematic review and meta-analysis. Sports 9, 126. 10.3390/sports9090126 34564331 PMC8471630

[B13] Grgic J. Pickering C. Bishop D. J. Schoenfeld B. J. Mikulic P. Pedisic Z. (2020b). CYP1A2 genotype and acute effects of caffeine on resistance exercise, jumping, and sprinting performance. J. Int. Soc. Sports. Nutr. 17, 21. 10.1186/s12970-020-00349-6 32295624 PMC7161272

[B14] Grgic J. Scapec B. Pedisic Z. Mikulic P. (2020a). Test-retest reliability of velocity and power in the deadlift and squat exercises assessed by the GymAware PowerTool system. Front. Physiol. 11, 561682. 10.3389/fphys.2020.561682 33013482 PMC7510176

[B15] Guest N. S. VanDusseldorp T. A. Nelson M. T. Grgic J. Schoenfeld B. J. Jenkins N. D. M. (2021). International society of sports nutrition position stand: caffeine and exercise performance. J. Int. Soc. Sports. Nutr. 18, 1. 10.1186/s12970-020-00383-4 33388079 PMC7777221

[B16] Jessen S. Eibye K. Christensen P. M. Hostrup M. Bangsbo J. (2021). No additive effect of acetaminophen when co-ingested with caffeine on cycling performance in well-trained young men. J. Appl. Physiol. 131, 238–249. 10.1152/japplphysiol.00108.2021 34013747

[B17] Küster M. Renner B. Oppel P. Niederweis U. Brune K. (2013). Consumption of analgesics before a marathon and the incidence of cardiovascular, gastrointestinal and renal problems: a cohort study. BMJ. Open 3, e002090. 10.1136/bmjopen-2012-002090 PMC3641448 23604350

[B18] Lixandrão M. E. Roschel H. Ugrinowitsch C. Miquelini M. Alvarez I. F. Libardi C. A. (2019). Blood-flow restriction resistance exercise promotes lower pain and ratings of perceived exertion compared with either high- or low-intensity resistance exercise performed to muscular failure. J. Sport. Rehabil. 28, 706–710. 10.1123/jsr.2018-0030 30040033

[B19] Mauger A. R. Jones A. M. Williams C. A. (2010). Influence of acetaminophen on performance during time trial cycling. J. Appl. Physiol. 108, 98–104. 10.1152/japplphysiol.00761.2009 19910336

[B20] Mauger A. R. Taylor L. Harding C. Wright B. Foster J. Castle P. C. (2014). Acute acetaminophen (paracetamol) ingestion improves time to exhaustion during exercise in the heat. Exp. Physiol. 99, 164–171. 10.1113/expphysiol.2013.075275 24058189

[B21] McLellan T. M. Caldwell J. A. Lieberman H. R. (2016). A review of caffeine's effects on cognitive, physical and occupational performance. Neurosci. Biobehav. Rev. 71, 294–312. 10.1016/j.neubiorev.2016.09.001 27612937

[B22] Morgan P. T. Bailey S. J. Banks R. A. Fulford J. Vanhatalo A. Jones A. M. (2019). Contralateral fatigue during severe-intensity single-leg exercise: influence of acute acetaminophen ingestion. Am. J. Physiol. Regul. Integr. Comp. Physiol. 317, R346-R354–R354. 10.1152/ajpregu.00084.2019 31141387 PMC6732432

[B23] Morgan P. T. Bowtell J. L. Vanhatalo A. Jones A. M. Bailey S. J. (2018). Acute acetaminophen ingestion improves performance and muscle activation during maximal intermittent knee extensor exercise. Eur. J. Appl. Physiol. 118, 595–605. 10.1007/s00421-017-3794-7 29332237 PMC5805811

[B24] Oliveira L. F. Saunders B. Yamaguchi G. Swinton P. Giannini A. G. (2020). Is individualization of sodium bicarbonate ingestion based on time to peak necessary? Med. Sci. Sports. Exerc. 52, 1801–1808. 10.1249/MSS.0000000000002313 32102054

[B25] Prescott L. F. (1980). Kinetics and metabolism of paracetamol and phenacetin. Br. J. Clin. Pharmacol. 10, 291S-298S–298S. 10.1111/j.1365-2125.1980.tb01812.x PMC1430174 7002186

[B26] Romdhani M. Souissi N. Dergaa I. Moussa-Chamari I. Chaabouni Y. Mahdouani K. (2022). The effect of caffeine, nap opportunity and their combination on biomarkers of muscle damage and antioxidant defence during repeated sprint exercise. Biol. Sport. 39, 1033–1042. 10.5114/biolsport.2023.112088 36247953 PMC9536395

[B27] Santos Junior E. R. T. de Salles B. F. Dias I. Ribeiro A. S. Simão R. Willardson J. M. (2021). Classification and determination model of resistance training status. Strength. Cond. J. 43, 77–86. 10.1519/ssc.0000000000000627

[B28] Saunders B. de Oliveira L. F. da Silva R. P. de Salles P. V. Gonçalves L. S. Yamaguchi G. (2017). Placebo in sports nutrition: a proof-of-principle study involving caffeine supplementation. Scand. J. Med. Sci. Sports 27, 1240–1247. 10.1111/sms.12793 27882605

[B29] Scapec B. Grgic J. Varovic D. Mikulic P. (2024). Caffeine, but not paracetamol (acetaminophen), enhances muscular endurance, strength, and power. J. Int. Soc. Sports. Nutr. 21, 2400513. 10.1080/15502783.2024.2400513 39246027 PMC11385662

[B30] Tomazini F. Santos-Mariano A. C. Andrade-Souza V. A. Sebben V. C. De Maria C. A. B. Coelho D. B. (2020). Caffeine but not acetaminophen increases 4-km cycling time-trial performance. PharmaNutrition 12, 100181. 10.1016/j.phanu.2020.100181

[B31] Varovic D. Grgic J. Schoenfeld B. J. Vuk S. (2023). Ergogenic effects of sodium bicarbonate on resistance exercise: a randomized, double-blind, placebo-controlled study. J. Strength. Cond. Res. 37, 1600–1608. 10.1519/JSC.0000000000004443 36752756

[B32] Whitcomb D. C. Block G. D. (1994). Association of acetaminophen hepatotoxicity with fasting and ethanol use. JAMA 272, 1845–1850. 10.1001/jama.1994.03520230055038 7990219

